# Endogenous endophthalmitis complicating *Streptococcus equi* subspecies *zooepidemicus* meningitis: a case report

**DOI:** 10.1186/s13104-015-1133-9

**Published:** 2015-05-05

**Authors:** Dominik Madžar, Mareike Hagge, Sebastian Möller, Martin Regensburger, De-Hyung Lee, Stefan Schwab, Jonathan Jantsch

**Affiliations:** Department of Neurology, Friedrich-Alexander-Universität (FAU) Erlangen-Nürnberg, Universitätsklinikum Erlangen, Schwabachanlage 6, 91054 Erlangen, Germany; Mikrobiologisches Institut – Klinische Mikrobiologie, Immunologie und Hygiene, Friedrich-Alexander-Universität (FAU) Erlangen-Nürnberg, Universitätsklinikum Erlangen, Wasserturmstraße 3-5, 91054 Erlangen, Germany; Present address: Institut für klinische Mikrobiologie und Hygiene, Universitätsklinikum Regensburg, Franz-Josef-Strauß-Allee 11, 93053 Regensburg, Germany

**Keywords:** Meningitis, Endophthalmitis, *Streptococcus equi* subspecies *zooepidemicus*

## Abstract

**Background:**

*Streptococcus equi* subspecies *zooepidemicus (Streptococcus zooepidemicus)* is a rare cause of meningitis in humans. Humans mainly get infected by contact with an animal source or by ingestion of unpasteurized dairy products. In rare cases, bacterial meningitis can be complicated by endogenous endophthalmitis which is frequently associated with a poor visual prognosis.

**Case presentation:**

A 73 year old male Caucasian patient presented with clinical signs indicative of bacterial meningitis. Blood and cerebrospinal fluid cultures yielded beta-hemolytic, catalase-negative cocci. The strain was identified as *Streptococcus zooepidemicus*. The patient was likely infected by contact with a sick horse. Under antibiotic treatment, his general condition improved rapidly. Early after hospital admission, however, he began seeing a black spot in his left eye’s central visual field. An ophthalmological examination revealed signs of endogenous endophthalmitis and so the patient underwent vitrectomy. Despite treatment, the visual acuity of his left eye remained severely impaired. He showed no further neurological deficits at hospital discharge.

**Conclusion:**

Meningitis caused by *Streptococcus zooepidemicus* is rare with only 27 previously published adult cases in the literature. Of note, this report constitutes the third description of endogenous endophthalmitis associated with *Streptococcus zooepidemicus* meningitis. Thus, endogenous endophthalmitis may represent a comparatively common complication of meningitis caused by this microorganism.

## Background

*Streptococcus equi* subspecies *zooepidemicus* (*S. zooepidemicus*) infections in humans are rare. Only 9 out of 308 isolates of group C streptococci cultured from clinical specimens represented *S. zooepidemicus* [1]. To date, a total of 27 adult patients have been reported who suffered from meningitis caused by *S. zooepidemicus* [2-8]. Most infections were linked to an animal source or inadequately pasteurized dairy products [2-7,9]. In addition, *S. zooepidemicus* can cause a wide variety of infections including sinusitis, endocarditis, septic arthritis and osteomyelitis [10], pericarditis [11], as well as streptococcal toxic shock syndrome [12].

## Case presentation

A 73 year old male Caucasian patient was admitted to the neurological intensive care unit because of fever, headache, neck stiffness, drowsiness, and general malaise. The patient had been well until two days before admission. Relevant comorbidities included coronary artery disease and a myocardial infarction three years ago. The patient was a retired farmer but still helped his son who had taken over the family business. Recently, he had been taking care of a sick horse suffering from an upper respiratory tract infection with purulent nasal discharge compatible with *coryza contagiosa equorum* (“strangles”). On physical examination, the patient was awake but confused and had nuchal rigidity. He had tachycardia with a heart rate of 126/min, fever (38.8°C), and an oxygen saturation of 96% while breathing 6 liters of oxygen/min. On auscultation of the chest, crackles were heard over both lungs. Chest radiography showed bilateral perihilar infiltrates. A systolic murmur was present at the right sternal border.

Results of routine laboratory tests showed leukocytopenia (3,580/μl; normal range 4,000-11,500/μl), thrombocytopenia (106,000/μl; normal range 160,000-400,000/μl), elevated C-reactive protein (116.9 mg/dl; normal <5 mg/dl) and procalcitonin levels (2.5 ng/ml; normal <0.5 ng/ml). A computed tomography scan of the head revealed no abnormalities. A lumbar puncture was performed. Cerebrospinal fluid (CSF) analysis yielded an elevated leukocyte count (575/μl, normal <4/μl), an elevated protein level (6,229 mg/dl, normal < 500 mg/dl), and an elevated lactate concentration (13.8 mmol/l, normal range 1.21-2.09 mmol/l). A CSF sample and blood cultures were taken for microbiological examination.

An antibiotic and antiviral regimen with ceftriaxone, ampicillin and aciclovir was initiated empirically. Furthermore, as an adjunctive treatment, glucocorticoids were administered (10 mg dexamethasone intravenously, 4×/d, for a total of 4 days).

**Figure 1:**
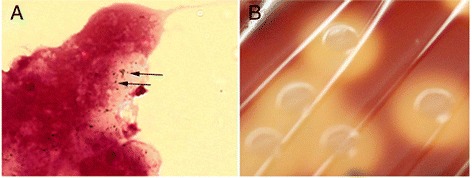
Gram-stain of cerebrospinal fluid specimen and colony morphology of *Streptococcus zooepidemicus* strain isolated from the patient. **(A)** Gram-stain of cerebrospinal fluid specimen. Arrows: Gram-positive cocci ordered in short chains. Magnification, x1000. **(B)** Colony morphology and appearance of *Streptococcus zooepidemicus* strain isolated from the patient grown on sheep blood agar plates.

Gram staining of the CSF specimen revealed neutrophils, cell debris and Gram-positive cocci laid out in (short) chains (Figure 1 A). Cultures yielded many large colonies of beta-hemolytic, catalase-negative cocci which were also detected in the blood cultures (Figure 1 B). The strain expressed the group C Lancefield antigen (Streptex; bioMérieux) and was identified as *S. zooepidemicus* by biochemical testing (API20 Strep, bioMérieux). The strain was sensitive to penicillin G, amoxicillin, ceftriaxone, erythromycin and vancomycin and resistant to clindamycin. A diagnosis of *S. zooepidemicus* meningitis was made. Based on these findings, aciclovir and ampicillin were discontinued and ceftriaxone was administered for a total of 21 days. After initiation of antibiotic therapy, the patient’s body temperature returned to normal levels within 24 hours. Transesophageal echocardiography revealed severe degenerative changes of the aortic valve with no signs suspicious of endocarditis, however. On the second day after admission, the patient reported to permanently see a black spot in his left eye’s central visual field. His visual acuity rapidly decreased to “hand motion” vision. An ophthalmological examination revealed signs of endophthalmitis and the patient eventually underwent vitrectomy. A vitreous specimen yielded no bacterial growth. Thereafter, his general condition improved steadily and he was discharged after a total of 21 days to a hospital specialized in rehabilitative care. On final examination, the only remaining neurological deficit was a markedly impaired visual acuity of his left eye.

## Conclusion

In our patient, the most probable source of infection was the close contact to an infected horse. It is likely that the streptococci primarily infested the airways and disseminated via the bloodstream into the meninges and the left eye. In line with our report, bacteremia was described in most cases of meningitis caused by *S. zooepidemicus*.

This case report constitutes the third description of endogenous endophthalmitis complicating meningitis caused by *S. zooepidemicus* [4,13]. Overall, endogenous endophthalmitis is generally considered a very rare complication of meningitis and bacteremia. However, it has now been reported in 10.7% of all published *S. zooepidemicus* meningitis cases. This may suggest it could occur more frequently in *S. zooepidemicus* meningitis than in meningitis caused by other microorganisms such as *Streptococcus pneumoniae* or *Neisseria meningitidis* [14,15]. Endogenous endophthalmitis is generally associated with poor visual outcome [16]. Early initiation of adequate therapy, including intravitreal injection of antibiotics or even vitrectomy, may preserve visual function [17]. Hence, watchful clinical observation of signs indicative of endophthalmitis is especially warranted in patients suffering from proven or suspected *S. zooepidemicus* meningitis, because, due to an altered mental status, these patients may not be able to verbalize physical complaints like loss of visual acuity.

### Consent

Written informed consent was obtained from the patient for publication of this Case Report and any accompanying images. A copy of the written consent is available for review by the Editor-in-Chief of this journal.

## Abbreviations

CSF: Cerebrospinal fluid
*S. zooepidemicus*: *Streptococcus equi* subspecies *zooepidemicus*
